# Uniqueness of Human Running Coordination: The Integration of Modern and Ancient Evolutionary Innovations

**DOI:** 10.3389/fpsyg.2016.00262

**Published:** 2016-04-11

**Authors:** John Kiely, David J. Collins

**Affiliations:** ^1^School of Health and Wellbeing, Institute of Coaching and Performance, University of Central LancashirePreston, UK; ^2^Fitness Department, Irish Rugby Football UnionDublin, Ireland

**Keywords:** evolution, sensoriomotor control, reflexes, preflexes, bio-tensegrity, practice-driven plasticity

## Abstract

Running is a pervasive activity across human cultures and a cornerstone of contemporary health, fitness, and sporting activities. Yet for the overwhelming predominance of human existence running was an essential prerequisite for survival. A means to hunt, and a means to escape when hunted. In a very real sense humans have evolved to run. Yet curiously, perhaps due to running's cultural ubiquity and the natural ease with which we learn to run, we rarely consider the uniqueness of human bipedal running within the animal kingdom. Our unique upright, single stance, bouncing running gait imposes a unique set of coordinative difficulties. Challenges demanding we precariously balance our fragile brains in the very position where they are most vulnerable to falling injury while simultaneously retaining stability, steering direction of travel, and powering the upcoming stride: all within the abbreviated time-frames afforded by short, violent ground contacts separated by long flight times. These running coordination challenges are solved through the tightly-integrated blending of primitive evolutionary legacies, conserved from reptilian and vertebrate lineages, and comparatively modern, more exclusively human, innovations. The integrated unification of these top-down and bottom-up control processes bestows humans with an agile control system, enabling us to readily modulate speeds, change direction, negotiate varied terrains and to instantaneously adapt to changing surface conditions. The seamless integration of these evolutionary processes is facilitated by pervasive, neural and biological, activity-dependent adaptive plasticity. Over time, and with progressive exposure, this adaptive plasticity shapes neural and biological structures to best cope with regularly imposed movement challenges. This pervasive plasticity enables the gradual construction of a robust system of distributed coordinated control, comprised of processes that are so deeply collectively entwined that describing their functionality in isolation obscures their true irrevocably entangled nature. Although other species rely on a similar set of coordinated processes to run, the bouncing bipedal nature of human running presents a specific set of coordination challenges, solved using a customized blend of evolved solutions. A deeper appreciation of the foundations of the running coordination phenomenon promotes conceptual clarity, potentially informing future advances in running training and running-injury rehabilitation interventions.

## Human running ability

Running is such a pervasive activity, across human cultures, that we often fail to appreciate how extraordinarily gifted we are as runners. We lack the swiftness of cheetahs; the power of charging bulls; the agility of cats. Yet we are exceptional running generalists, capable of running at moderate speeds for prolonged periods; readily modulating pace without changing fundamental gait pattern; seamlessly adapting to varying terrains and climatic conditions (Bramble and Lieberman, 2004).

Unlike other mammals who—thanks to embedded fixed action patterns and rapidly myelinating nervous systems—can quickly execute a limited repertoire of stereotypical movements, we remain helpless for prolonged periods after birth (Langen et al., 2011; Miller D. J. et al., 2012). This initial early life deficit, however, underpins a remarkable, slowly emerging coordinative proficiency. A proficiency, ultimately, enabling us to master a staggering diversity of skills unrivaled within the animal kingdom.

A sometimes overlooked distinction between running and more modern sporting movements is that running has been essential for survival across the expanse of hominid evolution (Bramble and Lieberman, 2004). How we run is shaped by our anatomy, neurology, and physiology. Yet, in a mutually reciprocating manner, how our long line of hominid ancestors once ran similarly contributed to sculpting current structural, neurological, and biological characteristics (Bramble and Lieberman, 2004). Throughout the countless blind “trial and error” experimental iterations of evolutionary deep-time, the mutually entangled co-evolution of bio-structures and running ability has led to the creation of deeply integrated coordinative solutions to the running challenge.

Despite the ubiquity of running within human cultures, and the everyday use of the term “coordination” within sporting domains, the running coordination phenomenon remains vaguely explored, perhaps overlooked as a key facilitator of our species unique running abilities.

Conventionally, movement coordination is viewed through the lens of one of a number of competing theories—Dynamical Systems; Equilibrium Point Hypothesis; Optimal Feedback Control—which exhibit both substantial overlap, and points of distinction: each variously explaining many, but not all, observable behaviors (Todorov, 2004). Discerning between these theories, as they apply to running coordination, is beyond the scope of this article and we instead focus on describing how modern and ancient evolutionary innovations blend to underpin human running performance.

### The evolutionary purpose of coordination

Evolutionary survival demands that biological systems—operating in unpredictable environments using unreliable components and finite energy sources—are robust to the challenges to which they are most commonly exposed (Kitano, 2004). In evolutionary terms the “threat,” imposed by running, takes many forms. If energy depletes; if mechanical tissue tolerances are exceeded; if neural processes are overloaded to the extent that movement precision and/or cognitive clarity declines, then inevitably, survival probability diminishes (Todorov, 2004; Niven and Laughlin, 2008; Skoyles, 2008; Miller R. H. et al., 2012). No single imperative necessarily predominates in any given context. Instead the neurobiological system seeks to satisfactorily and simultaneously resolve multiple partially-overlapping, yet partially-competing, organizational constraints (Wolpert et al., 2011).

## The running robustness challenge

Singularly within the mammalian kingdom, humans favor a prolonged upright, bouncing, bipedal running gait. Although other primates are capable of running for short distances, they are highly inefficient and hence reluctant runners (Bramble and Lieberman, 2004). We, however, run in an inherently unstable bouncing gait; managing impacts of multiple times bodyweight; steering direction of travel; retaining stability; generating sufficient propulsive forces to facilitate vigorous rearrangement of limb positions in preparation for upcoming contact: all within the abbreviated timeframes afforded by short ground collisions, interspersed between long flight periods.

Further complicating the running challenge are inevitable signal transmission and processing delays, in feedback and feedforward communication loops: delays impeding the rapidity with which the motor system can formulize, and action, responses to arising sensory information (Wolpert et al., 2011). Additionally, the dynamic multi-limb, multi-muscle nature of running produces unavoidable sensory “noise”: discrepancies between intended and actual muscle activations, errors in predicting behaviors of fragile soft-tissues, mis-estimations of characteristics of the external environment (Skoyles, 2006; Wolpert and Flanagan, 2010; Wolpert et al., 2011). All factors, theoretically, conspiring to ensure bipedal running is precariously unpredictable, and energetically and computationally expensive. Nevertheless, despite these apparent limitations, human running exhibits remarkable robustness under diversely challenging conditions.

## Highly-evolved top-down coordinated control of human running

From an evolutionary perspective upright locomotion appears a bizarre survival strategy: precariously balancing our fragile brains over the narrow base of support provided by our disproportionately skinny feet—in the very position where they are most vulnerable to falling injury. Our ability to safely run, in such an apparently dangerous manner, is facilitated by a comparatively recent evolutionary innovation: an innovation facilitated by our uniquely, in comparison to all other mammals, expanded cerebello-cerebral cortical circuitry (Todorov, 2004; Skoyles, 2008; Wolpert et al., 2011). Specifically, this cortical expansion has dramatically enhanced our ability to construct high-fidelity, temporally-resolved internal models capable of accurately predicting the likely consequences of upcoming interactions between body and external environment (Skoyles, 2006, 2008; Wolpert et al., 2011). This predictive capacity enhances anticipation of potential sources of upcoming perturbation, and underpins our ability to pre-prepare, and seamlessly integrate, advance-planned multi-level compensatory postural adjustments—customized to repel potential destabilizations—into on-going movement instructions (Skoyles, 2008).

We take such abilities for granted. Yet reliably predicting the future consequences of on-going muscle activations is a highly evolved complex task: requiring accurate estimation of current kinetics and kinematics, relative tissue behaviors and capacities, and the likely reactions of fallible bio-composite tissue structures to the shock load imposed by violent contact with a surface of uncertain integrity. This predictive ability demands the painstaking construction—over the course of our extensively prolonged maturation—of highly-detailed, experientially-driven internal models (Skoyles, 2008). Once matured these models permit the skillfully blending of sensory-informed estimates of current internal and external conditions, and accurate forecasting of upcoming de-stabilizations, to shape the emergence of the multi-level anticipatory postural adjustments necessary to preserve stability in the face of dynamically shifting running conditions (Skoyles, 2006; Wolpert and Flanagan, 2010; Wolpert et al., 2011).

### Multi-sensory cross-correlated mapping

As we repeatedly activate muscles, and receive sensory feedback on subsequent movement consequences, cross-correlated correspondences are gradually formed and refined: correspondences mapping the relationships between movement intentions, expectations, activations, and outcomes (Skoyles, 2006; Wolpert and Flanagan, 2010; Wolpert et al., 2011). Driven by persistent repetition, these practice acquired relationships capture, in detail, the integrated relationships between activation and sensation. Gradually, this constant triangulation of intention, activation, and sensory information drives a detailed mapping of the multi-dimensional sensorimotor landscape.

Slowly, with continued practice, discrepancies between projections and outcomes are progressively resolved: accuracy, sensitivity, and efficacy of activation strategy become ever-more finely calibrated. Ultimately these elaborately detailed internal models enable us to virtually simulate upcoming interactions, between runner and environment, and to formulise advance-planned remedial solutions (Todorov, 2004; Skoyles, 2006; Wolpert et al., 2011).

Consequently, when we run, the primary motor commands initiating and directing movement are accompanied by activation instructions, in the form of anticipatory feedforward motor adjustments, tailored to counteract forecasted upcoming destabilizations. Hence anticipated perturbations can be skillfully offset by the active orchestration of multiple potential micro-movement permutations managed in a centrally determined, precisely timed manner (Skoyles, 2008; Shadmehr et al., 2010; Wolpert et al., 2011).

The accuracy of perturbation predictions is dictated by the refined interpretation of emerging sensory information enabled by these richly-detailed practice-acquired models (Shadmehr et al., 2010; Wolpert and Flanagan, 2010). Ultimately, our evolved capacity to construct elaborative internal models underpins the skilled anticipation, and efficient remediation, of looming de-stabilizations. Thereby enabling the CNS to sensitively, rather than clumsily, calibrate micro-movement adjustments to best fit emerging context and offsetting the need for periodic gross emergency corrections: reducing energy cost, minimizing discomfort and offsetting injury risk. The calibrated clarity of these mapped relationships dynamically modifies in response to shifting circumstance: chronically, in response to factors such as practice-induced learning and accumulative neural or peripheral “wear-and-tear” and acutely, in response to mounting fatigue, soreness's and sensitivities (Wolpert et al., 2011).

### Spinally-mediated control

#### Central pattern generators: enhancing processing efficiency

Locomotion, in terrestrial and marine life-forms, is characterized by automated, cyclical patterns of muscle activation. Critical to the fluent execution of rhythmical gaits are spinally-located neural networks, Central Pattern Generators (CPG's; Thoroughman and Shadmehr, 2000; Lacquaniti et al., 2012). CPG's contain, embedded within their neural architecture, the undulating rhythmical patterns of motor neuron firing necessary to drive cyclical locomotive behaviors (Thoroughman and Shadmehr, 2000; Lacquaniti et al., 2012; Dzeladini et al., 2014). Although experimental difficulties remain a barrier to full understanding of CPG's in humans, recent work highlights their importance in evolutionary-prioritized gaits (Thoroughman and Shadmehr, 2000; Lacquaniti et al., 2012; Dzeladini et al., 2014).

The out-sourcing of evolutionary-critical activation templates to spinal CPG's economizes information storage and signal transmission efficiency: providing a means through which sparsely detailed low-dimensional inputs can be translated into coordinated patterns of richly-detailed, high-dimensional rhythmic outputs (Thoroughman and Shadmehr, 2000; Dzeladini et al., 2014). Thereby unburdening higher cortical centers from having to meticulously specify routine rhythmical activation patterns. Hence CPG's dramatically reduce the need for highly elaborative descending commands, from supra-spinal to spinal centers: minimizing precious communications bandwidth and providing a mechanism through which higher-cortical centers, rather than micro-managing movement specifics, need only fulfill an overseeing function (Thoroughman and Shadmehr, 2000). Once initiated CPG's are capable of autonomously sustaining locomotive activity, even switching between gaits with minimal descending guidance (Thoroughman and Shadmehr, 2000; Lacquaniti et al., 2012; Dzeladini et al., 2014). However, supra-spinal direction and sensory feedback add the updated detail necessary to adapt motor performance to best fit current context (Thoroughman and Shadmehr, 2000; Sidhu et al., 2012; Dzeladini et al., 2014).

Accordingly, when running demands are predictable, higher cortical resources are spared, allowing supra-spinal centers to devote resource to cognition and “executive-level” decision-making. If, however, the coordinative challenge escalates—due to, for example, unpredictable surfaces or mounting fatigue—, descending top-down direction intervenes to context-specifically customize CPG activity (Zehr et al., 2007; Ijspeert, 2008; Sidhu et al., 2012). Accordingly top-down intervention is more necessary, and strongest, in unpredictable environments imposing severe, non-formulaic challenges (Suzuki et al., 2004; Slobounov et al., 2006; Jahfari et al., 2012).

#### Running and reflexes

An unknown number of reflexes proliferate brainstem and spinal cord, each driving perturbation stabilizing responses in the absence of top-down supra-spinal commands (Heng and de Leon, 2007; Wolpaw and Chen, 2009; Dimitriou, 2014). In recent decades it has become apparent that reflexes are more pervasive; more widely distributed; more adaptive to context; more fluidly integrated with and manipulated by higher-level processes and sensory feedback, than historically envisaged (Jahfari et al., 2012). Given their automated action, reflexes offer a supplement to supra-spinal control, providing a mechanism to speedily action remedial responses to arising sensory information.

Reflexes are conventionally categorized along a spectrum of response times. Long-loop reflexes are highly modifiable and, as repeat practice adjusts inter-neuronal bias, can be customized to favorably regulate gain between afferent inputs and motor outputs. Through such mechanisms, regularly encountered movement permutations, of timings and positional cues, can be programmed to accentuate or dampen activation thresholds and response magnitudes: depending upon whether reflex activation helps or hinders desired movement outcomes (Heng and de Leon, 2007; Wolpaw and Chen, 2009; Dimitriou, 2014).

The stretch reflex, a reaction provoked when muscle spindles are suddenly stretched, serves as useful illustration. In comparison to non-runners, trained runners have readily triggered stretch reflexes, responding with heightened reflexive counter-actions (Ogawa et al., 2012). In contrast, ballet dancers, who habitually cushion ground reaction forces to finely control postures, substantially suppress stretch reflexes during practiced landing activities (Nielsen et al., 1993).

Unlike their more slowly responding longer-loop counterparts, fast-acting monosynaptic reflexes are less readily modifiable by experience (Wolpert and Flanagan, 2010). However, their inflexible reflexive reactions are predictable, and can therefore, with practice, be anticipated and productively integrated into movement plans.

### The inherent limitations of top-down neural control

Together these hierarchical neural processing modules, dispersed throughout supraspinal and spinal branches of the CNS, sensitively and responsively blend their collective outputs to direct running actions. There are, however, innate limitations to top-down neural control: inherent signal transmission delays in cortical communication and spinal reflex loops, unavoidable mis-estimations of tissue positioning's and capacities, unpredictable changes in surface integrity and impact conditions, and the ever-present sensory noise implicit in dynamic multi-limb, multi-tissue activity (Blickhan et al., 2006, 2013; Haeufle et al., 2012).

Such factors should, theoretically, greatly detract from the efficiency of bipedal running. Nevertheless, despite these apparent design flaws, the human neuro-mechanical system behaves remarkably proficiently when running. A proficiency ultimately facilitated by an incredibly ancient and primitive evolutionary innovation.

## Preflexes: evolution's movement-management shortcut

Intriguingly, when evolutionary-relevant impact activities—running, jumping—are closely scrutinized it appears evident that compensatory stabilizing reactions occur in advance of the fastest acting mono-synaptic reflexes (Brown and Loeb, 2000). Similarly when surprised by suddenly changing surface compliance, leg stiffness compensates in advance of altered EMG-signal: suggesting initial leg stiffness adjustments occur in the absence of top-down Instruction (Moritz and Farley, 2004; Daley et al., 2007; van der Krogt et al., 2009).

These mysterious instantaneous responses, as they occur “pre-reflexively,” have been termed “preflexes” and, as they operate without neural direction, are neither the same as, nor a sub-set of, reflexes (Brown and Loeb, 2000; Dickinson, 2000; Moritz and Farley, 2004). The preflex phenomenon is such an elegantly simple evolutionary innovation that its contribution to running remains, conventionally, overlooked.

### Solving the preflex puzzle: the bio-tensegrity solution

Over the span of evolutionary deep-time we have evolved from single cell entities, to dexterously skillful masters of our physical universe. At every step of this journey Nature's blind tinkering has persistently been pressurized by Darwinian imperatives to save energy; simplify control; avoid damage. Every dimension of our structural and material design has been shaped by these ever-present evolutionary imperatives: frequently leading to unexpected, highly innovative solutions to survival problems (for comprehensive review see Turvey and Fonseca, 2014).

The individual components of a tent become structurally stable only when the covering sheet is draped over a lattice of stiff poles, and appropriately tensioned by strategically placed lines and pegs. Biological organisms are, needless to remark, vastly more complex. Nevertheless, when scaled to the level of biological complexity, this generalized theme—whereby tensile and compressive components, when collectively pre-stressed in a specific configuration, exhibit disproportionate self-stabilizing resilience to deformation—appear a ubiquitous evolutionary innovation (Fuller, 1961; Ingber, 2008; Turvey and Fonseca, 2014). In engineering contexts, such configurations have been termed tensegrity systems (Fuller, 1961; Ingber, 2008). Experimental work, over the past three decades, reveals that molecules, cells, peripheral tissues, organs, and our entire bodies use such self-equilibrating design principles to repel suddenly imposed deformation: a phenomenon labeled bio-tensegrity (Levin, 2002; Schleip and Müller, 2013; Turvey and Fonseca, 2014).

At the level of the cell: actin microfilaments stiffen cell structures serving as conduits for mechanical stress; actomyosin microfilaments transmit forces continuously throughout the whole cell; intermediate microfilaments function as tensioned guide-wires stabilizing the cell. On the macro-scale, skeletal structures sustain compressive forces; muscle tissue generates contractile forces; the fascial web of connective tissue conveys tensile forces (Turvey and Fonseca, 2014).

The innate deformation-resistance, of any bio-tensegrity system, at the instant of load application, arises simply from the relative configuration of tensioning and stiffening elements and the structural integrity provided by a pervading binding pre-stress: a background “tone” strategically compressing stiff rod-like, and tensioning taut cable-like, elements of the system in a state of dynamic equilibrium.

This background pre-stress is not a product of neural activity, and is hence invisible to EMG (Turvey and Fonseca, 2014). Muscle, for example, has an electrically-invisible intrinsic “tone,” ensuring tissue is never completely lax. Similarly, the collagen lattice, of the all-encasing fascial network, imparts a tensioned structural integrity: binding and stiffening bundles of tissues through a unifying pre-stressed tautness (Schleip and Müller, 2013; Turvey and Fonseca, 2014). Through this pre-stressed medium each tissue cell is bound to the next. A mechanical deformation in one is instantaneously transmitted to its neighbor: ultimately scaling upwards to an all-enveloping, pre-tensioned connective web, unifying the mechanical state of each cell to that of the whole body (Levin, 2002; Schleip and Müller, 2013; Turvey and Fonseca, 2014).

Throughout the musculoskeletal system, tissues of the bio-composite connective net variously press and pull, stiffen and strain, against other tissues. Crucially, this arrangement is not haphazard but meticulously evolutionarily configured to resist, accommodate and productively harness the mechanical stresses and strains most pertinent to our species survival.

From micro- to macro-scales, our biological structures represent long series of nested bio-tensegrity systems capable of, individually and collectively, eliciting disproportionately non-linear restorative responses to imposed disruptions to biomechanical and bio-energetic homeostasis (Levin, 2002; Schleip and Müller, 2013; Turvey and Fonseca, 2014).

### The running bio-tensegrity system

During running, impact forces swirl in a multi-directional vortex: subjecting tissues to various degrees of compression, stretch and twist, as the shudder of impact reverberates through the system. To move safely, these forces must be dispersed to alleviate risks of exceeding critical tissue loading limits; to move efficiently, these forces must be channeled and re-deployed to optimally contribute to stabilization and propulsive power demands.

Positioning the leg in an orientation exploiting its innate material and geometrical properties creates conditions whereby the sudden shock of ground contact is safely absorbed and channeled for minimal effort, in terms of top-down direction and energetic demand. Driven by evolutionary imperatives and repeat practice, we progressively become more skilled at exploiting these built-in mechanical efficiencies. We gradually become more proficient at poising bio-tensegrity structures to more productively capitalize on “cheap” sources of control and propulsion merely by matching the physics of the situation to innate deformation-repelling design features (Brown and Loeb, 2000; Daley and Biewener, 2006; Biewener and Daley, 2007).

Furthermore, simply by leveraging properties of the mechanical system, the coordinated harnessing of our nested bio-tensegrity design remedies the inherent information-processing and perturbation-prediction deficits implicit in top-down control (Biewener and Daley, 2007).

**Figure 1:**
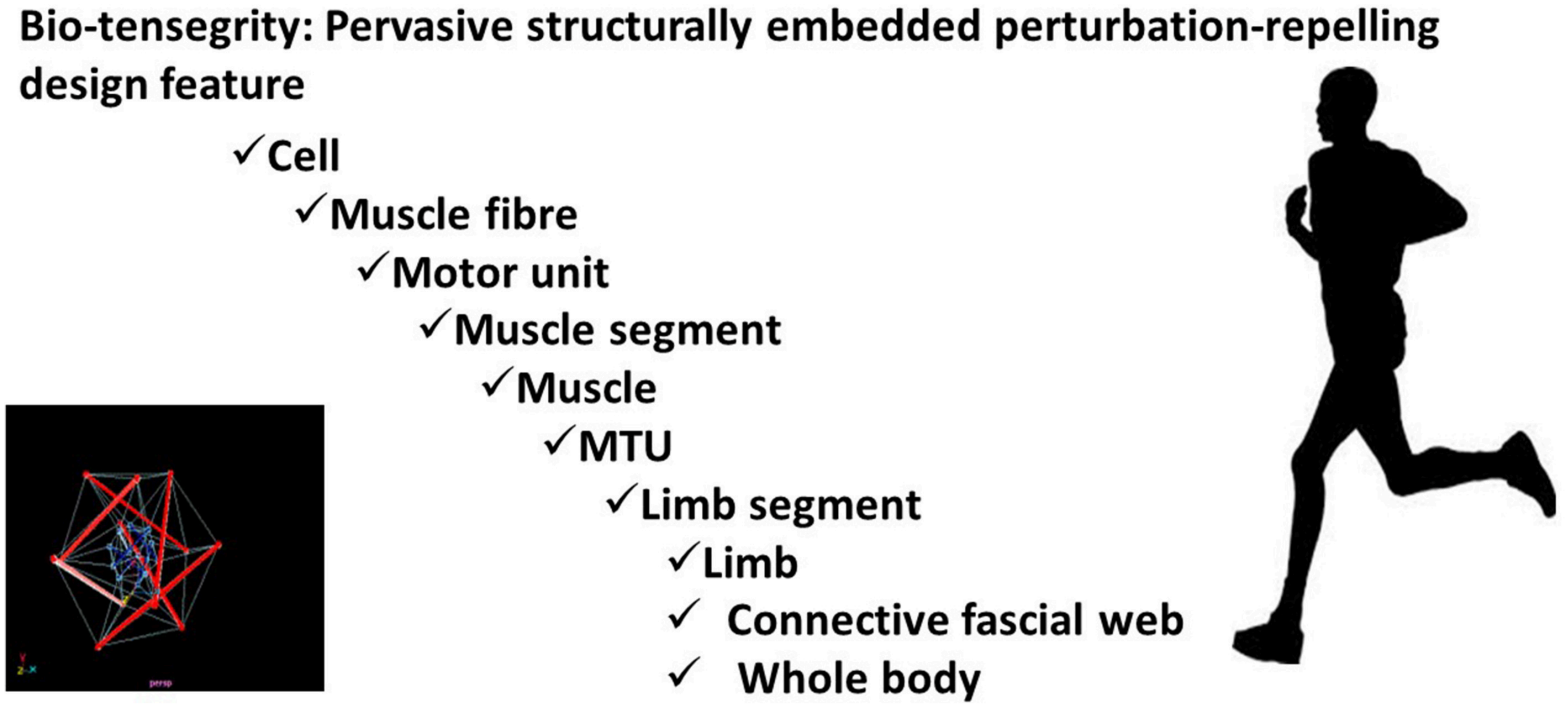
**Bio-tensegrity: Pervasive structurally embedded perturbation-repelling design feature**.

Thereby providing an instantaneous non-neurological, yet skilled, response to sudden perturbation: automatically buffering, stabilizing, re-directing, and re-cycling momentums for little energetic or neurological investment (See Figure 1).

## Running coordination: integration of the ancient and the new

### Practice-driven plasticity

This blending of archaic and comparatively recent evolutionary innovations is enabled by a pervasive characteristic of the human condition: activity-dependent plasticity. The capacity, both within the CNS and tissues of the periphery, to adapt—structurally, chemically, electrically, materially, and ultimately functionally—to repeated experience (Knikou, 2010; Taubert et al., 2010).

Throughout supra-spinal and spinal branches of the CNS persistent patterns of neural activations induce plastic re-configurations: modifications serving to micro-architecturally concretize relationships between regularly co-operating neural components, and between neuronal apparatus and activated motor units (Dickinson, 2006; Lemon, 2008; Taubert et al., 2010). Plasticity in the CNS is mirrored in the periphery, as tissues modify in response to habitual loading patterns. Muscle, in particular, is highly plastically evolvable: habitual loadings progressively sculpt the non-linear, visco-elastic, length-velocity-force relationships of muscular sub-compartments, thereby tailoring material and architectural characteristics to best fit regularly encountered movement contexts (Flück, 2006; Harridge, 2007; Hoppeler et al., 2011).

### Conclusion: coordinated blending of top-down and bottom-up control processes

The coordinated control of human running is enabled by the finely-tuned, tightly integrated blending of primitive evolutionary legacies, conserved from reptilian and vertebrate lineages, and comparatively modern, more exclusively human, innovations (Lemon, 2008). The operations of neuronal top-down, and mechanical bottom-up, control processes are so seamlessly integrated that describing their functionality in isolation is to obscure the true nature of coordinated running. There are no discontinuities where one ends and the other begins, instead organizational levels are irrevocably functionally entangled (Biewener and Daley, 2007).

**Figure 2:**
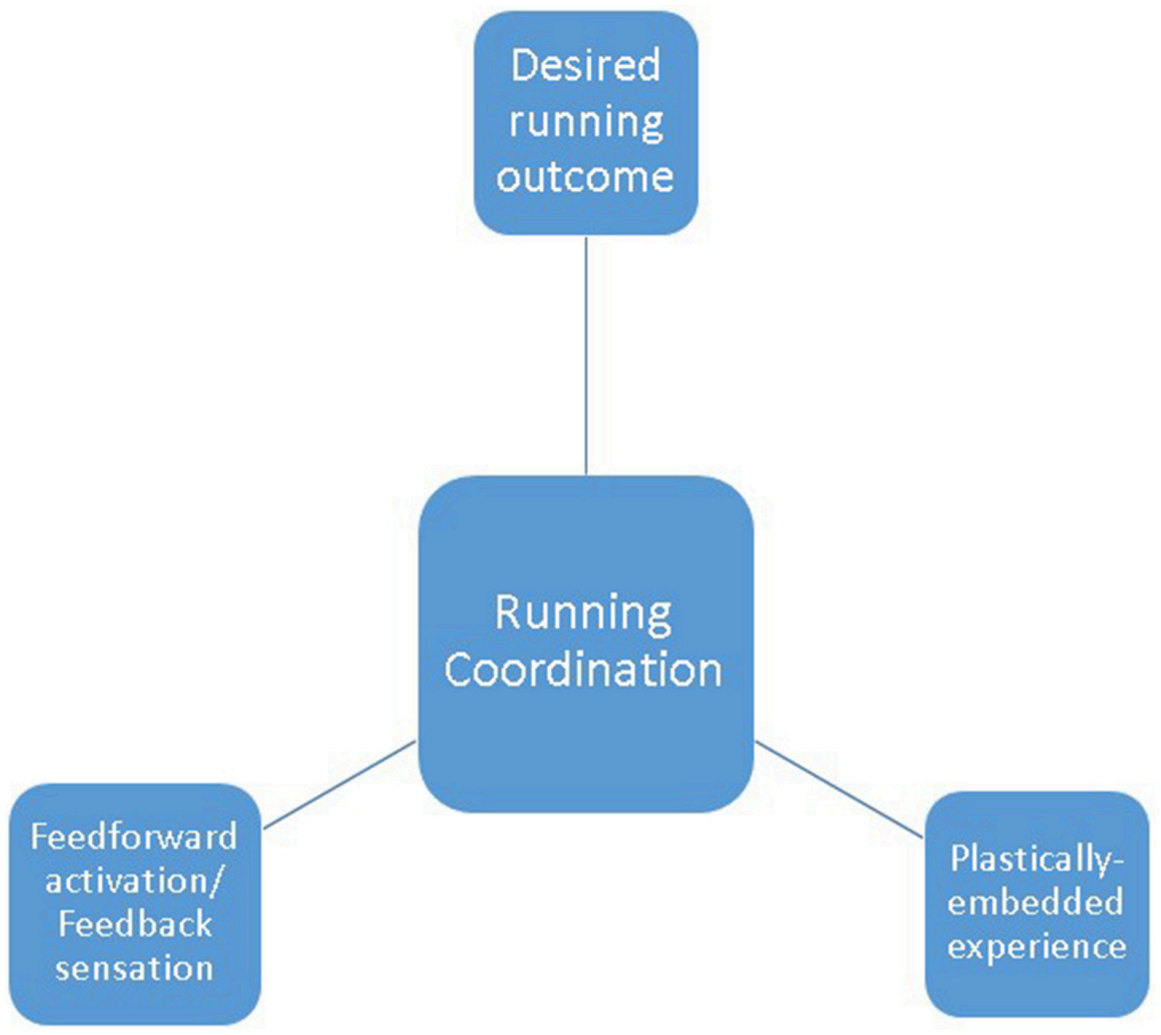
**Running coordination as the blend of plastically-embedded experience; sensorimotor integration of feedforward activation and feedback information; desired running outcome**.

When we run, top-down feedforward control responds to emerging multi-modal sensory information, to strategically orientate tissues to exploit our nested bio-tensegrity design (See Figure 2). On ground contact, immediate perturbation-buffering is provided by “dumb,” but skillfully manipulated, preflexive responses: dampening disturbances through tactical deployment of passive tissue properties; providing simple, but effective, control of imposed decelerations. As stance progresses, shorter-loop, then longer-loop reflexes are layered over initial preflexive responses, further customizing and supplementing control demands. Repetitive, cyclical activation patterns are delegated to spinally-located CPG's: reducing the control burden imposed on evolutionary-precious, energetically-costly supra-spinal centers (Todorov, 2004). The spinal cord thus serves, not as a rigidly hardwired communications conduit, but as a plastically modifying extension of higher neural centers: integrating CPG and reflex interactions with descending commands, and ascending sensory information (MacKay-Lyons, 2002). In the event of especially demanding coordinative challenge, higher-order neural resources intervene, exerting top-down executive direction: offsetting emerging instabilities by tailoring muscular activations to context-specific demands.

Over countless gait cycles, evolutionary-bestowed protective mechanisms persistently seek to extract more benefit, for less cost. We progressively learn to more astutely poise bio-composite tissue structures, in response to more sensitively interpreted sensory information (Haeufle et al., 2012).

This deeply integrated blending of strategies provides a robust system of collective, collaborative, distributed control. A system permeated with built-in overlapping degeneracies and compensatory fail-safes: enabling deficits, errors or failures from any control module, to be rescued by changing contributions from others (Whitacre, 2010; Mason, 2015). Ultimately enabling the human runner to negotiate varied challenges and terrains, for minimized neuronal investment, energetic cost and exposure to survival threatening trauma.

Without question the various coordinative undercurrents, incompletely summarized here, encompass complex unresolved academic puzzles. Further, such a conceptual understanding may, at first glance, seem far removed from real-World running performance and injury considerations. Importantly, however, blending these distinct strands provides a novel, insightful theoretical lens through which to conceptualize the underlying nature of human running coordination. A deeper conceptual appreciation, in turn, promoting the clarity necessary to drive future advances in running training and rehabilitation intervention designs.

Although other species similarly rely on coordinated processes to run, the unique demands imposed by the bouncing bipedal nature of human running presents a specific set of coordination challenges, solved using a novel configuration of evolved solutions.

## Author contributions

The author confirms being the sole contributor of this work and approved it for publication.

### Conflict of interest statement

The author declares that the research was conducted in the absence of any commercial or financial relationships that could be construed as a potential conflict of interest.
